# 4,5-Diferrocenyl-1,2-di­thiole-3-thione

**DOI:** 10.1107/S2414314622010112

**Published:** 2022-10-25

**Authors:** Jessica J. Sánchez-García, Marcos Flores-Alamo, Amairany Nuñez-Gordillo, Elena I. Klimova

**Affiliations:** a Facultad de Química, Universidad Nacional Autónoma de México, Ciudad Universitaria, Ciudad de México, 04510, Mexico; University Koblenz-Landau, Germany

**Keywords:** crystal structure, ferrocene, di­thiole-3-thione, inter­molecular contact

## Abstract

The structure of 4,5-diferrocenyl-1,2-di­thiole-3-thione, [Fe_2_(C_5_H_5_)_2_(C_13_H_8_S_3_)] or C_23_H_18_Fe_2_S_3_, at 130 K has monoclinic (*P*2_1_/*c*) symmetry. The molecule has two ferrocenyl units attached to a 1,2-di­thiole-3-thione moiety. It is of inter­est with respect to the question if the introduction of ferrocenyl substituents into biologically active mol­ecules offers the potential to obtain more efficacious therapeutic drugs. The crystal structure displays inter­molecular contacts of the C—H⋯S and S—π(C—C) types.

## Abstract 2

The structure of 4,5-diferrocenyl-1,2-di­thiole-3-thione at 130 K has monoclinic (*P*2_1_/*c*) symmetry. The compound has two ferrocenyl units attached to a 1,2-di­thiole-3-thione moiety. In the crystal array, there are inter­molecular contacts of the C—H⋯*S* and *S*—π(C–C) types.

## Graphical abstract

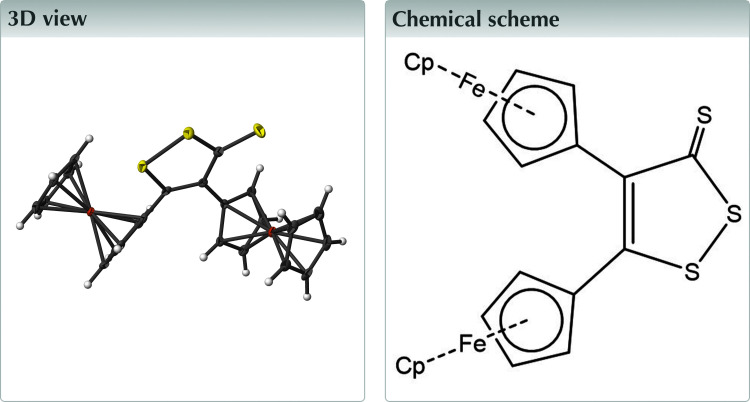

## Structure description

Ferrocene is known for its stable sandwich structure. The incorporation of ferrocene into biological mol­ecules offers the potential to develop better and more efficacious therapeutic drugs. 1,2-Di­thiole-3-thio­nes show significant biological activity, which include, amongst others, anti­tumour, anti­oxidant, chemotherapeutic, anti­thrombotic and radio-protective properties (Rakitin, 2021 ▸). The 1,2-di­thiole-3-thione moiety can be found in commercial drugs, such as Oltipraz (Maxuitenko *et al.*, 1998 ▸), anethole di­thiol­ethione ADT (Chen *et al.*, 2010 ▸), S-Danshensu (Bian *et al.*, 2012 ▸) and NOSH-1 (Jia *et al.*, 2013 ▸). The synthons can be useful for many sulfur heterocycles (Konstanti­nova *et al.*, 2007 ▸) and their optical properties have been employed for the creation of organic electronic conductors (Yamashita *et al.*, 1998 ▸), photoconductive materials (Perepichka *et al.*, 2001 ▸) and semiconducting polymers (Hou *et al.*, 2011 ▸).

**Figure 1:**
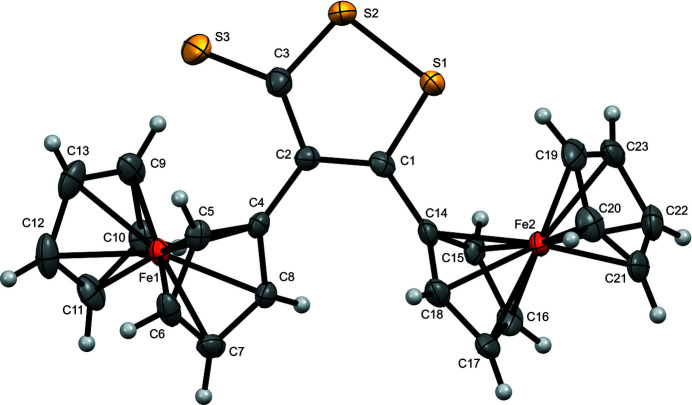
The mol­ecular structure of the title compound, with displacement ellipsoids drawn at the 70% probability level.

**Figure 2:**
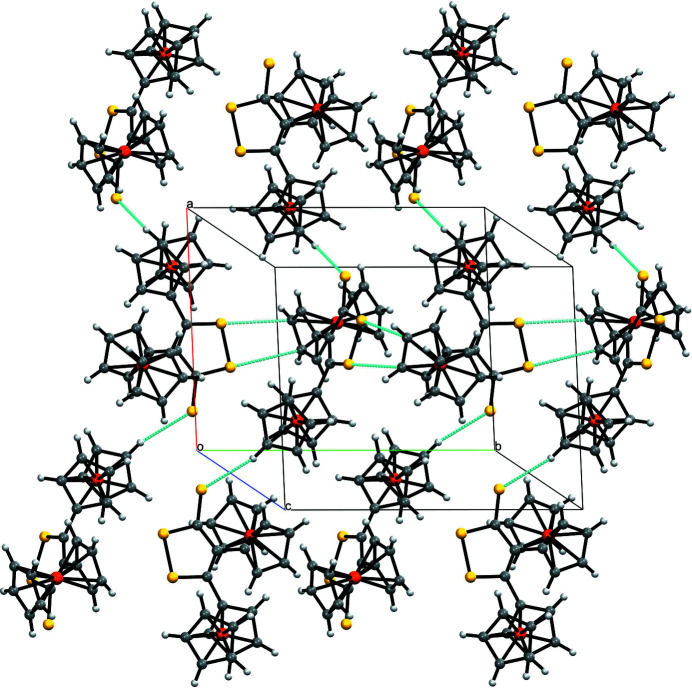
The crystal structure of the title compound along the base vector [010], showing the inter­molecular contacts of the S⋯π(C—C) type as dotted turquoise lines.

The asymmetric unit of the title compound is constituted by one mol­ecule showing two ferrocenyl units attached to a 1,2-di­thiole-3-thione ring (Fig. 1 ▸). The cyclo­penta­dienyl (Cp) rings bonded to the same Fe atom are almost parallel, with angles of 4.06 (2) and 4.24 (2)° between the Cp planes for the ferrocenyl groups of Fe1 and Fe2, respectively. In addition, the Cp rings of each ferrocenyl moiety adopt an eclipsed conformation. The 1,2-di­thiole-3-thione ring is planar, with an r.m.s. deviation of 0.0295 for the plane of the equation −3.79 (2)*x* + 9.17 (1)*y* + 10.04 (1)*z* = 4.21 (1). The angles between the 1,2-di­thiole-3-thione ring and the directly bonded Cp rings (C4–C8 and C14–C18) are 33.31 (3) and 48.16 (2)°. There is an inter­molecular C—H⋯S inter­action (C21—H21⋯S3) of 2.88 Å, with an angle of 139°. Moreover, another inter­molecular inter­action of the S⋯π(C—C) type between the S—S bond and an aromatic C—C bond of one of the Cp rings is observed (S1⋯C6 = 3.22 Å and S2⋯C7 = 3.45 Å) is observed. Fig. 2 ▸ shows a projection of the crystal structure approximately along [001]. In summary, the packing of the mol­ecules is assumed to be mainly dictated by van der Waals forces.

## Synthesis and crystallization

To a mixture of sodium sulfide (10 mmol) and S_8_ (10 mmol) in ethanol (80 ml) was added 1,2-diferrocenyl­cyclo­propenone (5 mmol) and the solution was stirred at 353 K for 8 h. After the solvent had been removed *in vacuo*, the resulting residue was purified by column chromatography with alumina using a mixture of hexane and diethyl ether (1:1 *v*/*v*). Black crystals of 4,5-diferrocenyl-1,2-di­thiole-3-thione suitable for single-crystal diffraction analysis were obtained by slow evaporation of a saturated di­chloro­methane/hexane (1:1 *v*/*v*) solution (yield 50%; m.p. 498–500 K).

^1^H NMR (400 MHz, CDCl_3_): δ 4.12 (5H, *s*, C_5_H_5_), 4.18 (5H, *s*, C_5_H_5_), 4.19 (2H, *m*, C_5_H_4_), 4.35 (2H, *m*, C_5_H_4_), 4.38 (2H, *m*, C_5_H_4_), 4.40 (2H, *m*, C_5_H_4_). ^13^C NMR (75 MHz, CDCl_3_): δ 67.45 (CH C_5_H_4_), 69.71 (C_5_H_5_), 69.74 (CH C_5_H_4_), 70.14 (CH C_5_H_4_), 70.92 (C_5_H_5_), 71.45 (CH C_5_H_4_), 79.60 (C_*ipso*_ C_5_H_4_), 80.05 (C_*ipso*_ C_5_H_4_), 141.37 (=C), 169.18 (=C), 214.00 (C=S). MS: *m*/*z* 502, [*M*]^+^ 40. Analysis calculated (%) for C_23_H_18_Fe_2_S_3_: C 55.02, H 3.61, S 19.15; found: C 55.10, H 3.71, S 19.22.

## Refinement

Crystal data, data collection and structure refinement details are summarized in Table 1 ▸.

## Supplementary Material

Crystal structure: contains datablock(s) global, I. DOI: 10.1107/S2414314622010112/im4016sup1.cif

Structure factors: contains datablock(s) I. DOI: 10.1107/S2414314622010112/im4016Isup2.hkl

Print cif. DOI: 10.1107/S2414314622010112/im4016sup3.pdf

CCDC reference: 2213735

Additional supporting information:  crystallographic information; 3D view; checkCIF report

## Figures and Tables

**Table 1:** Experimental details

Crystal data	
Chemical formula	[Fe_2_(C_5_H_5_)_2_(C_13_H_8_S_3_)]
*M*_r_	502.25
Crystal system, space group	Monoclinic, *P*2_1_/*c*
Temperature (K)	130
*a*, *b*, *c* (Å)	11.0149 (12), 14.0459 (12), 13.3983 (13)
β (°)	109.205 (12)
*V* (Å^3^)	1957.5 (4)
*Z*	4
Radiation type	Mo *K*α
μ (mm^−1^)	1.81
Crystal size (mm)	0.57 × 0.46 × 0.11

Data collection	
Diffractometer	Agilent Xcalibur Atlas Gemini
Absorption correction	Analytical (*CrysAlis RED*; Agilent, 2013 ▸)
*T*_min_, *T*_max_	0.486, 0.852
No. of measured, independent and observed [*I* > 2σ(*I*)] reflections	10068, 4559, 3445
*R*_int_	0.039
(sin θ/λ)_max_ (Å^−1^)	0.692

Refinement	
*R*[*F* ^2^ > 2σ(*F* ^2^)], *wR*(*F* ^2^), *S*	0.039, 0.079, 1.04
No. of reflections	4559
No. of parameters	253
H-atom treatment	H-atom parameters constrained
Δρ_max_, Δρ_min_ (e Å^−3^)	0.43, −0.41

Computer programs: *CrysAlis PRO* and *CrysAlis RED* (Agilent, 2013 ▸), *SHELXS2018* (Sheldrick, 2015*a* ▸), *SHELXL2018* (Sheldrick, 2015*b* ▸), *ORTEP-3 for Windows* (Farrugia, 2012 ▸) and *Mercury* (Macrae *et al.*, 2020 ▸).

## full crystallographic data

### Crystal data

**Table:**

[Fe_2_(C_5_H_5_)_2_(C_13_H_8_S_3_)]	*F*(000) = 1024
*M**_r_* = 502.25	*D*_x_ = 1.704 Mg m^−^^3^
Monoclinic, *P*2_1_/*c*	Mo *K*α radiation, λ = 0.71073 Å
Hall symbol: -P 2ybc	Cell parameters from 2429 reflections
*a* = 11.0149 (12) Å	θ = 3.5–29.5°
*b* = 14.0459 (12) Å	µ = 1.81 mm^−^^1^
*c* = 13.3983 (13) Å	*T* = 130 K
β = 109.205 (12)°	Plate, black
*V* = 1957.5 (4) Å^3^	0.57 × 0.46 × 0.11 mm
*Z* = 4	

### Data collection

**Table:**

Agilent Xcalibur Atlas Gemini diffractometer	4559 independent reflections
Graphite monochromator	3445 reflections with *I* > 2σ(*I*)
Detector resolution: 10.4685 pixels mm^-1^	*R*_int_ = 0.039
ω scans	θ_max_ = 29.5°, θ_min_ = 3.5°
Absorption correction: analytical (*CrysAlis RED*; Agilent, 2013)	*h* = −15→13
*T*_min_ = 0.486, *T*_max_ = 0.852	*k* = −19→14
10068 measured reflections	*l* = −16→18

### Refinement

**Table:**

Refinement on *F*^2^	0 restraints
Least-squares matrix: full	Hydrogen site location: inferred from neighbouring sites
*R*[*F*^2^ > 2σ(*F*^2^)] = 0.039	H-atom parameters constrained
*wR*(*F*^2^) = 0.079	*w* = 1/[σ^2^(*F*_o_^2^) + (0.023*P*)^2^ + 0.8521*P*] where *P* = (*F*_o_^2^ + 2*F*_c_^2^)/3
*S* = 1.04	(Δ/σ)_max_ < 0.001
4559 reflections	Δρ_max_ = 0.43 e Å^−^^3^
253 parameters	Δρ_min_ = −0.41 e Å^−^^3^

### Special details

**Table:**

Geometry. All esds (except the esd in the dihedral angle between two l.s. planes) are estimated using the full covariance matrix. The cell esds are taken into account individually in the estimation of esds in distances, angles and torsion angles; correlations between esds in cell parameters are only used when they are defined by crystal symmetry. An approximate (isotropic) treatment of cell esds is used for estimating esds involving l.s. planes.

### Fractional atomic coordinates and isotropic or equivalent isotropic displacement parameters (Å^2^)

**Table:**

	*x*	*y*	*z*	*U*_iso_*/*U*_eq_	
C1	0.4091 (3)	0.39934 (18)	0.2096 (2)	0.0128 (6)	
C2	0.5328 (3)	0.38193 (18)	0.2754 (2)	0.0128 (6)	
C3	0.6322 (3)	0.4379 (2)	0.2544 (2)	0.0173 (6)	
C4	0.5612 (2)	0.31407 (19)	0.3639 (2)	0.0124 (6)	
C5	0.6716 (3)	0.25355 (18)	0.4048 (2)	0.0144 (6)	
H5	0.74328	0.25135	0.380255	0.017*	
C6	0.6561 (3)	0.1977 (2)	0.4878 (2)	0.0181 (6)	
H6	0.715344	0.151857	0.528342	0.022*	
C7	0.5366 (3)	0.22197 (19)	0.5000 (2)	0.0179 (6)	
H7	0.50179	0.195138	0.549818	0.021*	
C8	0.4783 (3)	0.29337 (19)	0.4246 (2)	0.0147 (6)	
H8	0.397726	0.322723	0.415683	0.018*	
C9	0.7111 (3)	0.4770 (2)	0.5497 (2)	0.0194 (7)	
H9	0.699657	0.524867	0.497316	0.023*	
C10	0.6239 (3)	0.4545 (2)	0.6037 (2)	0.0177 (6)	
H10	0.543627	0.484821	0.593797	0.021*	
C11	0.6767 (3)	0.3790 (2)	0.6750 (2)	0.0213 (7)	
H11	0.638054	0.349734	0.72102	0.026*	
C12	0.7972 (3)	0.3551 (2)	0.6656 (2)	0.0244 (7)	
H12	0.853732	0.306978	0.704488	0.029*	
C13	0.8192 (3)	0.4150 (2)	0.5880 (2)	0.0238 (7)	
H13	0.892609	0.414117	0.565643	0.029*	
C14	0.2895 (3)	0.35130 (19)	0.2068 (2)	0.0140 (6)	
C15	0.1742 (3)	0.3995 (2)	0.2065 (2)	0.0156 (6)	
H15	0.162618	0.466391	0.20834	0.019*	
C16	0.0809 (3)	0.3289 (2)	0.2032 (2)	0.0209 (7)	
H16	−0.003988	0.340458	0.203319	0.025*	
C17	0.1353 (3)	0.2384 (2)	0.1995 (2)	0.0209 (7)	
H17	0.092877	0.17904	0.196154	0.025*	
C18	0.2636 (3)	0.2511 (2)	0.2016 (2)	0.0176 (6)	
H18	0.322191	0.201926	0.199956	0.021*	
C19	0.1889 (3)	0.3340 (2)	−0.0603 (2)	0.0208 (7)	
H19	0.27249	0.347447	−0.062494	0.025*	
C20	0.1376 (3)	0.2426 (2)	−0.0567 (2)	0.0197 (7)	
H20	0.180714	0.183946	−0.055911	0.024*	
C21	0.0111 (3)	0.2533 (2)	−0.0543 (2)	0.0201 (7)	
H21	−0.04585	0.203153	−0.051976	0.024*	
C22	−0.0160 (3)	0.3527 (2)	−0.0560 (2)	0.0205 (7)	
H22	−0.094107	0.380448	−0.054727	0.025*	
C23	0.0941 (3)	0.4027 (2)	−0.0601 (2)	0.0201 (7)	
H23	0.102955	0.469885	−0.062245	0.024*	
Fe1	0.65338 (4)	0.33776 (3)	0.52362 (3)	0.01289 (11)	
Fe2	0.13851 (4)	0.31869 (3)	0.07340 (3)	0.01267 (11)	
S1	0.38303 (7)	0.48864 (5)	0.11639 (6)	0.01651 (16)	
S2	0.57355 (7)	0.52584 (5)	0.15958 (6)	0.01945 (17)	
S3	0.79134 (7)	0.43455 (6)	0.30660 (7)	0.0298 (2)	

### Atomic displacement parameters (Å^2^)

**Table:**

	*U*^11^	*U*^22^	*U*^33^	*U*^12^	*U*^13^	*U*^23^
C1	0.0184 (14)	0.0122 (13)	0.0083 (13)	−0.0008 (11)	0.0049 (12)	−0.0026 (11)
C2	0.0153 (14)	0.0121 (13)	0.0112 (13)	−0.0026 (11)	0.0048 (12)	−0.0021 (11)
C3	0.0166 (14)	0.0213 (15)	0.0137 (14)	−0.0011 (12)	0.0044 (12)	−0.0015 (13)
C4	0.0112 (13)	0.0131 (13)	0.0116 (13)	−0.0025 (11)	0.0020 (11)	−0.0033 (12)
C5	0.0159 (14)	0.0118 (14)	0.0142 (14)	0.0005 (11)	0.0033 (12)	−0.0049 (12)
C6	0.0222 (16)	0.0124 (14)	0.0139 (14)	0.0024 (12)	−0.0019 (13)	−0.0014 (12)
C7	0.0227 (16)	0.0153 (14)	0.0143 (14)	−0.0074 (13)	0.0042 (13)	0.0004 (12)
C8	0.0121 (14)	0.0176 (15)	0.0123 (14)	−0.0036 (12)	0.0014 (12)	−0.0007 (12)
C9	0.0273 (16)	0.0136 (14)	0.0138 (14)	−0.0035 (13)	0.0018 (13)	−0.0043 (12)
C10	0.0209 (15)	0.0171 (15)	0.0135 (14)	0.0040 (12)	0.0033 (12)	−0.0076 (12)
C11	0.0293 (17)	0.0231 (16)	0.0090 (14)	0.0015 (14)	0.0029 (13)	−0.0036 (13)
C12	0.0226 (16)	0.0236 (17)	0.0182 (16)	0.0058 (14)	−0.0052 (13)	−0.0077 (14)
C13	0.0171 (16)	0.0279 (17)	0.0228 (16)	−0.0058 (13)	0.0016 (13)	−0.0127 (15)
C14	0.0145 (14)	0.0160 (14)	0.0083 (13)	0.0002 (12)	−0.0005 (11)	0.0012 (12)
C15	0.0150 (14)	0.0217 (15)	0.0082 (13)	−0.0019 (12)	0.0010 (12)	−0.0047 (12)
C16	0.0170 (15)	0.0377 (19)	0.0094 (13)	−0.0040 (14)	0.0061 (12)	−0.0037 (14)
C17	0.0213 (16)	0.0284 (17)	0.0104 (14)	−0.0082 (14)	0.0015 (13)	0.0049 (13)
C18	0.0206 (15)	0.0150 (15)	0.0137 (14)	−0.0014 (12)	0.0010 (12)	0.0012 (12)
C19	0.0207 (16)	0.0318 (18)	0.0088 (13)	−0.0042 (14)	0.0033 (12)	−0.0029 (13)
C20	0.0248 (16)	0.0210 (16)	0.0125 (14)	0.0025 (13)	0.0051 (13)	−0.0048 (13)
C21	0.0200 (15)	0.0247 (16)	0.0105 (14)	−0.0069 (13)	−0.0020 (12)	−0.0014 (13)
C22	0.0156 (15)	0.0280 (17)	0.0132 (14)	0.0013 (13)	−0.0016 (12)	−0.0006 (13)
C23	0.0244 (17)	0.0195 (15)	0.0112 (14)	−0.0047 (13)	−0.0011 (13)	0.0032 (13)
Fe1	0.0142 (2)	0.0121 (2)	0.0100 (2)	0.00071 (16)	0.00085 (17)	−0.00160 (16)
Fe2	0.0116 (2)	0.0164 (2)	0.00860 (19)	−0.00166 (16)	0.00152 (16)	0.00006 (17)
S1	0.0167 (4)	0.0158 (4)	0.0143 (3)	−0.0020 (3)	0.0014 (3)	0.0036 (3)
S2	0.0181 (4)	0.0207 (4)	0.0178 (4)	−0.0060 (3)	0.0035 (3)	0.0045 (3)
S3	0.0138 (4)	0.0402 (5)	0.0334 (5)	−0.0021 (4)	0.0051 (4)	0.0137 (4)

### Geometric parameters (Å, º)

**Table:**

C1—C2	1.379 (4)	C12—H12	0.95
C1—C14	1.470 (4)	C13—Fe1	2.055 (3)
C1—S1	1.726 (3)	C13—H13	0.95
C2—C3	1.449 (4)	C14—C18	1.433 (4)
C2—C4	1.472 (4)	C14—C15	1.438 (4)
C3—S3	1.661 (3)	C14—Fe2	2.053 (3)
C3—S2	1.736 (3)	C15—C16	1.418 (4)
C4—C5	1.436 (4)	C15—Fe2	2.040 (3)
C4—C8	1.439 (4)	C15—H15	0.95
C4—Fe1	2.072 (3)	C16—C17	1.412 (4)
C5—C6	1.417 (4)	C16—Fe2	2.042 (3)
C5—Fe1	2.046 (3)	C16—H16	0.95
C5—H5	0.95	C17—C18	1.416 (4)
C6—C7	1.420 (4)	C17—Fe2	2.041 (3)
C6—Fe1	2.028 (3)	C17—H17	0.95
C6—H6	0.95	C18—Fe2	2.048 (3)
C7—C8	1.418 (4)	C18—H18	0.95
C7—Fe1	2.033 (3)	C19—C20	1.410 (4)
C7—H7	0.95	C19—C23	1.422 (4)
C8—Fe1	2.045 (3)	C19—Fe2	2.053 (3)
C8—H8	0.95	C19—H19	0.95
C9—C10	1.415 (4)	C20—C21	1.412 (4)
C9—C13	1.428 (4)	C20—Fe2	2.041 (3)
C9—Fe1	2.051 (3)	C20—H20	0.95
C9—H9	0.95	C21—C22	1.426 (4)
C10—C11	1.418 (4)	C21—Fe2	2.040 (3)
C10—Fe1	2.043 (3)	C21—H21	0.95
C10—H10	0.95	C22—C23	1.418 (4)
C11—C12	1.415 (4)	C22—Fe2	2.047 (3)
C11—Fe1	2.043 (3)	C22—H22	0.95
C11—H11	0.95	C23—Fe2	2.063 (3)
C12—C13	1.419 (5)	C23—H23	0.95
C12—Fe1	2.050 (3)	S1—S2	2.0525 (10)
			
C2—C1—C14	128.6 (2)	C20—C21—C22	107.8 (3)
C2—C1—S1	119.1 (2)	C20—C21—Fe2	69.79 (16)
C14—C1—S1	112.35 (19)	C22—C21—Fe2	69.84 (16)
C1—C2—C3	115.4 (2)	C20—C21—H21	126.1
C1—C2—C4	122.2 (2)	C22—C21—H21	126.1
C3—C2—C4	122.3 (2)	Fe2—C21—H21	125.9
C2—C3—S3	131.5 (2)	C23—C22—C21	108.0 (3)
C2—C3—S2	113.9 (2)	C23—C22—Fe2	70.40 (16)
S3—C3—S2	114.56 (17)	C21—C22—Fe2	69.32 (15)
C5—C4—C8	106.3 (2)	C23—C22—H22	126
C5—C4—C2	128.3 (3)	C21—C22—H22	126
C8—C4—C2	125.4 (2)	Fe2—C22—H22	125.8
C5—C4—Fe1	68.61 (15)	C22—C23—C19	107.5 (3)
C8—C4—Fe1	68.57 (15)	C22—C23—Fe2	69.22 (17)
C2—C4—Fe1	129.04 (19)	C19—C23—Fe2	69.44 (16)
C6—C5—C4	108.7 (3)	C22—C23—H23	126.2
C6—C5—Fe1	68.97 (16)	C19—C23—H23	126.2
C4—C5—Fe1	70.56 (15)	Fe2—C23—H23	126.7
C6—C5—H5	125.6	C6—Fe1—C7	40.94 (12)
C4—C5—H5	125.6	C6—Fe1—C11	120.22 (12)
Fe1—C5—H5	126.4	C7—Fe1—C11	104.35 (13)
C5—C6—C7	108.3 (2)	C6—Fe1—C10	156.95 (13)
C5—C6—Fe1	70.32 (16)	C7—Fe1—C10	121.46 (12)
C7—C6—Fe1	69.73 (16)	C11—Fe1—C10	40.62 (11)
C5—C6—H6	125.9	C6—Fe1—C8	68.57 (11)
C7—C6—H6	125.9	C7—Fe1—C8	40.69 (11)
Fe1—C6—H6	125.7	C11—Fe1—C8	120.94 (12)
C8—C7—C6	107.9 (3)	C10—Fe1—C8	107.83 (11)
C8—C7—Fe1	70.12 (15)	C6—Fe1—C5	40.71 (11)
C6—C7—Fe1	69.33 (16)	C7—Fe1—C5	68.62 (12)
C8—C7—H7	126.1	C11—Fe1—C5	157.56 (11)
C6—C7—H7	126.1	C10—Fe1—C5	161.08 (11)
Fe1—C7—H7	126.1	C8—Fe1—C5	68.41 (11)
C7—C8—C4	108.9 (2)	C6—Fe1—C12	105.52 (12)
C7—C8—Fe1	69.19 (15)	C7—Fe1—C12	119.71 (12)
C4—C8—Fe1	70.53 (15)	C11—Fe1—C12	40.44 (12)
C7—C8—H8	125.6	C10—Fe1—C12	68.02 (12)
C4—C8—H8	125.6	C8—Fe1—C12	156.00 (13)
Fe1—C8—H8	126.3	C5—Fe1—C12	123.14 (12)
C10—C9—C13	107.9 (3)	C6—Fe1—C9	159.73 (13)
C10—C9—Fe1	69.51 (16)	C7—Fe1—C9	159.11 (12)
C13—C9—Fe1	69.81 (16)	C11—Fe1—C9	68.25 (12)
C10—C9—H9	126.1	C10—Fe1—C9	40.44 (12)
C13—C9—H9	126.1	C8—Fe1—C9	125.06 (11)
Fe1—C9—H9	126.2	C5—Fe1—C9	125.45 (12)
C9—C10—C11	108.3 (3)	C12—Fe1—C9	68.12 (12)
C9—C10—Fe1	70.06 (16)	C6—Fe1—C13	122.13 (12)
C11—C10—Fe1	69.68 (16)	C7—Fe1—C13	156.59 (12)
C9—C10—H10	125.8	C11—Fe1—C13	68.25 (13)
C11—C10—H10	125.8	C10—Fe1—C13	68.21 (12)
Fe1—C10—H10	126	C8—Fe1—C13	162.09 (12)
C12—C11—C10	107.8 (3)	C5—Fe1—C13	109.31 (12)
C12—C11—Fe1	70.04 (18)	C12—Fe1—C13	40.45 (13)
C10—C11—Fe1	69.70 (16)	C9—Fe1—C13	40.70 (12)
C12—C11—H11	126.1	C6—Fe1—C4	68.90 (11)
C10—C11—H11	126.1	C7—Fe1—C4	68.94 (11)
Fe1—C11—H11	125.8	C11—Fe1—C4	158.45 (12)
C11—C12—C13	108.4 (3)	C10—Fe1—C4	124.24 (11)
C11—C12—Fe1	69.52 (16)	C8—Fe1—C4	40.90 (11)
C13—C12—Fe1	69.96 (17)	C5—Fe1—C4	40.83 (10)
C11—C12—H12	125.8	C12—Fe1—C4	160.70 (12)
C13—C12—H12	125.8	C9—Fe1—C4	110.39 (11)
Fe1—C12—H12	126.3	C13—Fe1—C4	125.76 (12)
C12—C13—C9	107.5 (3)	C21—Fe2—C15	149.91 (12)
C12—C13—Fe1	69.59 (17)	C21—Fe2—C20	40.48 (12)
C9—C13—Fe1	69.49 (16)	C15—Fe2—C20	169.40 (12)
C12—C13—H13	126.2	C21—Fe2—C17	104.53 (12)
C9—C13—H13	126.2	C15—Fe2—C17	68.54 (12)
Fe1—C13—H13	126.3	C20—Fe2—C17	114.90 (12)
C18—C14—C15	107.5 (2)	C21—Fe2—C16	115.13 (12)
C18—C14—C1	127.9 (3)	C15—Fe2—C16	40.64 (11)
C15—C14—C1	124.6 (2)	C20—Fe2—C16	147.95 (12)
C18—C14—Fe2	69.38 (15)	C17—Fe2—C16	40.47 (12)
C15—C14—Fe2	68.97 (15)	C21—Fe2—C22	40.84 (11)
C1—C14—Fe2	126.0 (2)	C15—Fe2—C22	118.59 (12)
C16—C15—C14	107.5 (3)	C20—Fe2—C22	68.24 (12)
C16—C15—Fe2	69.75 (16)	C17—Fe2—C22	126.44 (12)
C14—C15—Fe2	69.89 (16)	C16—Fe2—C22	107.48 (12)
C16—C15—H15	126.2	C21—Fe2—C18	125.60 (11)
C14—C15—H15	126.2	C15—Fe2—C18	68.99 (12)
Fe2—C15—H15	125.7	C20—Fe2—C18	106.65 (12)
C17—C16—C15	108.6 (3)	C17—Fe2—C18	40.51 (11)
C17—C16—Fe2	69.72 (17)	C16—Fe2—C18	68.30 (12)
C15—C16—Fe2	69.60 (16)	C22—Fe2—C18	163.99 (11)
C17—C16—H16	125.7	C21—Fe2—C14	165.36 (11)
C15—C16—H16	125.7	C15—Fe2—C14	41.13 (11)
Fe2—C16—H16	126.6	C20—Fe2—C14	129.44 (12)
C16—C17—C18	108.6 (3)	C17—Fe2—C14	68.42 (11)
C16—C17—Fe2	69.82 (17)	C16—Fe2—C14	68.46 (11)
C18—C17—Fe2	70.02 (17)	C22—Fe2—C14	153.54 (11)
C16—C17—H17	125.7	C18—Fe2—C14	40.92 (11)
C18—C17—H17	125.7	C21—Fe2—C19	67.94 (12)
Fe2—C17—H17	126	C15—Fe2—C19	132.43 (12)
C17—C18—C14	107.8 (3)	C20—Fe2—C19	40.29 (12)
C17—C18—Fe2	69.47 (16)	C17—Fe2—C19	149.68 (12)
C14—C18—Fe2	69.70 (15)	C16—Fe2—C19	169.72 (12)
C17—C18—H18	126.1	C22—Fe2—C19	67.94 (12)
C14—C18—H18	126.1	C18—Fe2—C19	118.68 (12)
Fe2—C18—H18	126.3	C14—Fe2—C19	111.23 (12)
C20—C19—C23	108.3 (3)	C21—Fe2—C23	68.23 (12)
C20—C19—Fe2	69.38 (17)	C15—Fe2—C23	111.25 (12)
C23—C19—Fe2	70.14 (17)	C20—Fe2—C23	68.04 (12)
C20—C19—H19	125.8	C17—Fe2—C23	165.92 (12)
C23—C19—H19	125.8	C16—Fe2—C23	130.29 (12)
Fe2—C19—H19	126.2	C22—Fe2—C23	40.38 (12)
C19—C20—C21	108.3 (3)	C18—Fe2—C23	153.47 (12)
C19—C20—Fe2	70.33 (17)	C14—Fe2—C23	121.35 (11)
C21—C20—Fe2	69.73 (17)	C19—Fe2—C23	40.42 (12)
C19—C20—H20	125.8	C1—S1—S2	93.98 (10)
C21—C20—H20	125.8	C3—S2—S1	97.18 (10)
Fe2—C20—H20	125.7		
			
C14—C1—C2—C3	176.4 (3)	Fe1—C9—C13—C12	−59.4 (2)
S1—C1—C2—C3	−4.0 (3)	C10—C9—C13—Fe1	59.30 (19)
C14—C1—C2—C4	−6.1 (4)	C2—C1—C14—C18	−51.1 (4)
S1—C1—C2—C4	173.5 (2)	S1—C1—C14—C18	129.3 (3)
C1—C2—C3—S3	−174.6 (2)	C2—C1—C14—C15	130.3 (3)
C4—C2—C3—S3	7.9 (4)	S1—C1—C14—C15	−49.3 (3)
C1—C2—C3—S2	7.0 (3)	C2—C1—C14—Fe2	−141.9 (2)
C4—C2—C3—S2	−170.5 (2)	S1—C1—C14—Fe2	38.5 (3)
C1—C2—C4—C5	146.3 (3)	C18—C14—C15—C16	1.0 (3)
C3—C2—C4—C5	−36.4 (4)	C1—C14—C15—C16	179.8 (2)
C1—C2—C4—C8	−31.3 (4)	Fe2—C14—C15—C16	59.86 (19)
C3—C2—C4—C8	146.0 (3)	C18—C14—C15—Fe2	−58.88 (19)
C1—C2—C4—Fe1	−121.3 (3)	C1—C14—C15—Fe2	120.0 (3)
C3—C2—C4—Fe1	56.0 (4)	C14—C15—C16—C17	−1.0 (3)
C8—C4—C5—C6	0.1 (3)	Fe2—C15—C16—C17	59.0 (2)
C2—C4—C5—C6	−177.9 (3)	C14—C15—C16—Fe2	−59.95 (19)
Fe1—C4—C5—C6	58.56 (19)	C15—C16—C17—C18	0.6 (3)
C8—C4—C5—Fe1	−58.48 (18)	Fe2—C16—C17—C18	59.5 (2)
C2—C4—C5—Fe1	123.5 (3)	C15—C16—C17—Fe2	−58.91 (19)
C4—C5—C6—C7	0.1 (3)	C16—C17—C18—C14	0.0 (3)
Fe1—C5—C6—C7	59.64 (19)	Fe2—C17—C18—C14	59.40 (19)
C4—C5—C6—Fe1	−59.53 (18)	C16—C17—C18—Fe2	−59.4 (2)
C5—C6—C7—C8	−0.3 (3)	C15—C14—C18—C17	−0.6 (3)
Fe1—C6—C7—C8	59.76 (19)	C1—C14—C18—C17	−179.4 (3)
C5—C6—C7—Fe1	−60.01 (19)	Fe2—C14—C18—C17	−59.3 (2)
C6—C7—C8—C4	0.3 (3)	C15—C14—C18—Fe2	58.62 (19)
Fe1—C7—C8—C4	59.56 (19)	C1—C14—C18—Fe2	−120.2 (3)
C6—C7—C8—Fe1	−59.26 (19)	C23—C19—C20—C21	0.1 (3)
C5—C4—C8—C7	−0.2 (3)	Fe2—C19—C20—C21	59.6 (2)
C2—C4—C8—C7	177.8 (2)	C23—C19—C20—Fe2	−59.53 (19)
Fe1—C4—C8—C7	−58.74 (19)	C19—C20—C21—C22	−0.3 (3)
C5—C4—C8—Fe1	58.50 (17)	Fe2—C20—C21—C22	59.7 (2)
C2—C4—C8—Fe1	−123.5 (3)	C19—C20—C21—Fe2	−59.99 (19)
C13—C9—C10—C11	−0.1 (3)	C20—C21—C22—C23	0.3 (3)
Fe1—C9—C10—C11	59.39 (19)	Fe2—C21—C22—C23	60.0 (2)
C13—C9—C10—Fe1	−59.49 (19)	C20—C21—C22—Fe2	−59.7 (2)
C9—C10—C11—C12	0.3 (3)	C21—C22—C23—C19	−0.3 (3)
Fe1—C10—C11—C12	59.9 (2)	Fe2—C22—C23—C19	59.07 (19)
C9—C10—C11—Fe1	−59.62 (19)	C21—C22—C23—Fe2	−59.4 (2)
C10—C11—C12—C13	−0.4 (3)	C20—C19—C23—C22	0.1 (3)
Fe1—C11—C12—C13	59.3 (2)	Fe2—C19—C23—C22	−58.9 (2)
C10—C11—C12—Fe1	−59.69 (19)	C20—C19—C23—Fe2	59.06 (19)
C11—C12—C13—C9	0.3 (3)	C2—C1—S1—S2	−0.3 (2)
Fe1—C12—C13—C9	59.36 (19)	C14—C1—S1—S2	179.37 (19)
C11—C12—C13—Fe1	−59.1 (2)	C2—C3—S2—S1	−6.2 (2)
C10—C9—C13—C12	−0.1 (3)	S3—C3—S2—S1	175.17 (15)

## References

[bb1] Agilent (2013). *CrysAlis PRO* and *CrysAlis RED*. Agilent Technologies, Yarnton, Oxfordshire, England.

[bb2] Bian, J., Cai, Z. & Wu, H. (2012). CN Patent 102417501 A, 1.

[bb3] Chen, P., Luo, Y., Hai, L., Qian, S. & Wu, Y. (2010). *Eur. J. Med. Chem.* **45**, 3005–3010. 10.1016/j.ejmech.2010.03.029 20392547

[bb4] Farrugia, L. J. (2012). *J. Appl. Cryst.* **45**, 849–854.

[bb5] Hou, Y., Long, G., Sui, D., Cai, Y., Wan, X., Yu, A. & Chen, Y. (2011). *Chem. Commun.* **47**, 10401–10403. 10.1039/c1cc13511b 21842048

[bb6] Jia, J., Xiao, Y., Wang, W., Qing, L., Xu, Y., Song, H., Zhen, X., Ao, G., Alkayed, N. J. & Cheng, J. (2013). *Neurochem. Int.* **62**, 1072–1078. 10.1016/j.neuint.2013.04.001 PMC4040256 23587562

[bb7] Konstantinova, L. S., Berezin, A. A., Lysov, K. A. & Rakitin, O. A. (2007). *Tetrahedron Lett.* **48**, 5851–5854.

[bb8] Macrae, C. F., Sovago, I., Cottrell, S. J., Galek, P. T. A., McCabe, P., Pidcock, E., Platings, M., Shields, G. P., Stevens, J. S., Towler, M. & Wood, P. A. (2020). *J. Appl. Cryst.* **53**, 226–235. 10.1107/S1600576719014092 PMC6998782 32047413

[bb9] Maxuitenko, Y., Libby, A. H., Joyner, H. H., Curphey, T. J., MacMillan, D. L., Kensler, T. W. & Roebuck, B. D. (1998). *Carcinogenesis*, **19**, 1609–1615. 10.1093/carcin/19.9.1609 9771932

[bb10] Perepichka, D. F., Perepichka, I. F., Bryce, M. R., Moore, A. J. & Sokolov, N. I. (2001). *Synth. Met.* **121**, 1487–1488.

[bb11] Rakitin, O. A. (2021). *Molecules*, **26**, 3595–3638. 10.3390/molecules26123595 PMC8231234 34208356

[bb12] Sheldrick, G. M. (2015*a*). *Acta Cryst.* A**71**, 3–8.

[bb14] Sheldrick, G. M. (2015*b*). *Acta Cryst.* C**71**, 3–8.

[bb13] Yamashita, Y., Tomura, M. & Badruz Zaman, M. (1998). *Chem. Commun.* pp. 1657–1658.

