# Supplementary material for: 4,5-Diferrocenyl-1,2-di­thiole-3-thione
Source: IUCrdata. 2022 Oct 25;7(Pt 10):x221011. doi: 10.1107/S2414314622010112 (PMC9638058; doi:10.1107/S2414314622010112)
Supplement: Supplementary file 3 [file x-07-x221011-sup3.pdf]

4,5-diferrocenyl-1,2-dithiol-3-thione

Jessica J. Sánchez-García, Marcos Flores-Alamo, Amairany Nuñez-Gordillo and Elena I. Klimova*

Facultad de Química, Universidad Nacional Autónoma de México, Ciudad Universitaria, Ciudad de México, 04510, Mexico

Correspondence email: eiklimova@yahoo.com.mx

Abstract

The structure of 4,5-diferrocenyl-1,2-dithiol-3-thione, $C_{23}H_{18}Fe_2S_3$, at 130 K has monoclinic ($P2_1/c$) symmetry. The compound has two ferrocenyl units bridged by a 3-dithiol-3-thione moiety. It is of interest with respect to the ferrocenyl into biological molecules offers the potential to develop better more efficacious therapeutic drugs. The crystal the packing is assumed to be mainly dictated by van der Waals forces.

Structure description

Ferrocene is known for its stable sandwich structure. The incorporation of ferrocenyl into biological molecules offers the potential to develop better and more efficacious therapeutic drugs. 1,2-dithiole-3-thiones show significant biological activity, which include amongst others antitumor, antioxidant, chemotherapeutic, antithrombotic and radio protective properties (Rakitin *et al.*, 2021), The 1,2-dithiole-3-thione moiety can be found in commercial drugs such as Oltipraz (Maxuitenko 15 *et al.*, 1998) anethole dithiolethione ADT (Chen *et al.*, 2010), S-Danshensu (Bian *et al.*, 2012) and NOSH-1 (Jia *et al.*, 16 2013). The synthons can be useful for many sulfurs heterocycles (Konstantinova *et al.*, 2007), and their optical properties have been employed for the creation of organic electronic conductors (Yamashita *et al.*, 1998), photoconductive materials (Perepichka *et al.*, 2001), and semiconducting polymers (Hou *et al.*, 2011).

The asymmetric unit of the title compound is constituted by a pair of ferrocenyl units bridged by a dithiol-3-thione ring (Fig. 1). The cyclopentadienyl (Cp) rings are almost parallel, with angle of 4.06 (2) and 4.24 (2)° between Cp planes for ferrocenyl groups with Fe1 and Fe2 respectively. The cyclopentadienyl rings of the ferrocenyl moiety adopt eclipsed conformation. The dithiol-3-thione ring is planarity with r.m.s. of 0.0295 for $-$ 3.79 (2) x + 9.17 (1) y + 10.04 (1) z = 4.21 (1) equation plane. The dithiol-3-thione ring, form a angle of 34.91 (2) and 49.20 (8)°, with C4/C8 and C14/C18 rings respectively. The dihedral angles between the dithiol-3-thione ring and the substituted cyclopentadienyl rings are 36.42 (3) and 49.28 (2)°. The crystalline array, it is stabilized by the weak intermolecular interactions C—H····S, S—S···π mainly. The intermolecular C21—H21····S3 contact is found at 2.883 Å, while the interaction S—S···π it is set between the dithiol-3-thione group and the cyclopentadienyl (Cp) ring of the ferrocenyl group. The figure 2 shows the [010] projection of the unit cell. In summary, the packing of the molecules is assumed to be mainly dictated by van der Waals forces.

Synthesis and crystallization

Mixture of sodium sulfide (10 mmol) and S_8 (10 mmol) in ethanol (80 ml) is added the 1,2-diferrocenylcyclopropenone (5 mmol) was stirred at 353 K for 8 h. The solvents were removed *in vacuo*, the residues was purified by column chromatography with alumina, and hexane: diethyl ether (ratio 1:1 *v*/*v*). crystals of 4,5-diferrocenyl-1,2-dithiol-3-thione, suitable for single-crystal diffraction analysis, were obtained by slow evaporation of saturated dichloromethane/hexane (ratio 1:1 *v*/*v*) solution, yield (50%), black crystals, mp. 498–500 K.

^{1}H NMR(Fig. 3) (400 MHz, $CDCl_3$) δ: 4.12 (5 H, s, C_5H_5), δ: 4.18 (5 H, s, C_5H_5), δ: 4.19 (2 H, m, C_5H_4), δ: 4.35 (2 H, m, C_5H_4), δ: 4.38 (2 H, m, C_5H_4), δ: 4.40 (2 H, m, C5H4) p.p.m.,^{13}C NMR (Fig. 4) (75 MHz, $CDCl_3$) δ: 69.71, 70.92 (C_5H_5), 67.45, 69.74, 70.14, 70.92 ($2C_5H_4$) 79.60, 80.05 (Cipso Fc), 141.37, 169.18 (C), 214.0 (C=S) p.p.m., MS: m/*z* 502 [*M*]+ 40. Anal. Calcd for $C_{23}H_{18}Fe_2S_3$: C, 55.02, H, 3.61, S, 19.15, Found C, 55.10, H, 3.71, S, 19.22%.

Refinement details

Crystal data, data collection and structure refinement details are summarized in Table 1.

Table 1

Experimental details

Crystal data	
Chemical formula	$C_{23}H_{18}Fe_2S_3$
M_r	502.25
Crystal system, space group	Monoclinic, $P2_1/c$
Temperature (K)	130
a, b, c (Å)	11.0149 (12), 14.0459 (12), 13.3983 (13)
β (°)	109.205 (12)
V (Å^3)	1957.5 (4)
Z	4
Radiation type	Mo $K\alpha$
μ (mm^{-1})	1.81
Crystal size (mm)	0.57 × 0.46 × 0.11
Data collection	
Diffractometer	Xcalibur, Atlas, Gemini
Absorption correction	Analytical (*CrysAlis RED*; Agilent, 2013)
T_{min}, T_{max}	0.486, 0.852
No. of measured, independent and observed [$I > 2\sigma(I)$] reflections	10068, 4559, 3445
R_{int}	0.039
$(\sin \theta/\lambda)_{max}$ (Å^{-1})	0.692
Refinement	
$R[F^2 > 2\sigma(F^2)]$, $wR(F^2)$, S	0.039, 0.079, 1.04
No. of reflections	4559
No. of parameters	253
H-atom treatment	H-atom parameters constrained
$\Delta\rho_{max}$, $\Delta\rho_{min}$ (e Å^{-3})	0.43, −0.41

Computer programs: *CrysAlis PRO*; Agilent, 2013, *CrysAlis PRO*; Agilent, 2013, *CrysAlis RED*; Agilent, 2013, *SHELXS2018* (Sheldrick, 2015), *SHELXL2018* (Sheldrick, 2015), *ORTEP-3 for Windows* (Farrugia, 2012), *Mercury* (Macrae *et al.*, 2008).

Acknowledgements

The authors thank PAPIIT-DGAPA-UNAM (**IN 217421)** for their financial support of this work.

Funding information

Funding for this research was provided by: The authors thank PAPIIT-DGAPA-UNAM (IN 217421) for their financial support of this work .

References

Agilent (2013). *CrysAlis PRO* and *CrysAlis RED*. Agilent Technologies, Yarnton Oxfordshire, England

Bian, J., Cai, Z. & Wu, H. (2012). *CN Patent* 102417501 A, 1

Chen, P., Luo, Y., Hai, L., Qian, S. & Wu, Y. (2010). *Eur. J. Med. Chem*, **45**, 3005–3010

Hou, Y., Long, G., Sui, D., Cai, Y., Wan, X., Yu, A. & Chen, Y. (2011). *Chem. Commun*, **47**, 10401–10403

Jia, J., Xiao, Y., Wang, W., Wang, L., Xu, Y., Song, H., Zhen, X., Ao, G., Alksasyed, N. & Cheng, J. (2013). Neurochem. Int. 62, 1072–1078

Konstantinova, L. S., Berezin, A. A., Lysov, K. A. & Rakitin, O. A. (2007). *Tetrahedron Lett*. **48**, 5851-5854

Maxuitenko, Y., Libby, A. H., Joyner, H. H., Curphey, T. J., MacMillan, D. L., Kensler, T. W. & Roebuck, B. D. (1998). Carcinogenesis, 19, 1609–1615

Perepichka, D. F., Perepichka, I. F., Bryce, M. R., Moore, A. J. & Sokolov, N. I. (2001). *Synth. Met*. **121**, 1487–1488

Rakitin, O. A. (2021). Molecules, 26 (12), 3595-3638

Sheldrick, G. M. (2015). *Acta Cryst.* A**71**, 3–8.

Sheldrick, G. M. (2015). *Acta Cryst.* C**71**, 3–8.

Yamashita, Y., Tomura, M. & Zaman, M. B. (1998). Chem. Commun. 1657-1658

Figure 1

The *ORTEP* diagram of the compound **4,5-diferrocenyl-1,2-dithiol-3-thione**. Displacement ellipsoids are drawn at the 70% probability level.

Figure 2

The crystal array of the title compound showing intermolecular contacts of the type C—H···*S* and *S*···\p, along the base vector [010].

Figure 3

1*H*-NMR (400 MHz, CDCl3, TMS) spectrum of compound 4,5-diferrocenyl-1,2-dithiol-3-thione.

Figure 4

13 C-NMR (100 MHz, CDCl3, TMS) spectrum of compound 4,5-diferrocenyl-1,2-dithiol-3-thione

full crystallographic data

4,5-diferrocenyl-1,2-dithiol-3-thione

(ke0822)

Crystal data

$C_{23}H_{18}Fe_2S_3$
M_r = 502.25
Monoclinic, $P2_1/c$
Hall symbol: -P 2ybc
a = 11.0149 (12) Å
b = 14.0459 (12) Å
c = 13.3983 (13) Å
β = 109.205 (12)°
V = 1957.5 (4) Å^3
Z = 4

F(000) = 1024
D_x = 1.704 Mg m^{-3}
Mo $K\alpha$ radiation, λ = 0.71073 Å
Cell parameters from 2429 reflections
θ = 3.5–29.5°
μ = 1.81 mm^{-1}
T = 130 K
Plate, black
0.57 × 0.46 × 0.11 mm

Data collection

Xcalibur, Atlas, Gemini diffractometer
Graphite monochromator
Detector resolution: 10.4685 pixels mm^{-1}
ω scans
Absorption correction: analytical (*CrysAlis RED*; Agilent, 2013)
T_{min} = 0.486, T_{max} = 0.852

10068 measured reflections
4559 independent reflections
3445 reflections with $I > 2\sigma(I)$
R_{int} = 0.039
θ_{max} = 29.5°, θ_{min} = 3.5°
h = −15→13
k = −19→14
l = −16→18

Refinement

Refinement on F^2
Least-squares matrix: full
$R[F^2 > 2\sigma(F^2)]$ = 0.039
$wR(F^2)$ = 0.079
S = 1.04
4559 reflections
253 parameters
0 restraints

Hydrogen site location: inferred from neighbouring sites
H-atom parameters constrained
$w = 1/[\sigma^2(F_o^2) + (0.023P)^2 + 0.8521P]$ where $P = (F_o^2 + 2F_c^2)/3$
$(\Delta/\sigma)_{max}$ < 0.001
$\Delta\rho_{max}$ = 0.43 e Å^{-3}
$\Delta\rho_{min}$ = −0.41 e Å^{-3}

Special details

Geometry. All e.s.d.'s (except the e.s.d. in the dihedral angle between two l.s. planes) are estimated using the full covariance matrix. The cell e.s.d.'s are taken into account individually in the estimation of e.s.d.'s in distances, angles and torsion angles; correlations between e.s.d.'s in cell parameters are only used when they are defined by crystal symmetry. An approximate (isotropic) treatment of cell e.s.d.'s is used for estimating e.s.d.'s involving l.s. planes.

Fractional atomic coordinates and isotropic or equivalent isotropic displacement parameters (Å^2)

	x	y	z	U_{iso}*/U_{eq}
C1	0.4091 (3)	0.39934 (18)	0.2096 (2)	0.0128 (6)
C2	0.5328 (3)	0.38193 (18)	0.2754 (2)	0.0128 (6)
C3	0.6322 (3)	0.4379 (2)	0.2544 (2)	0.0173 (6)
C4	0.5612 (2)	0.31407 (19)	0.3639 (2)	0.0124 (6)
C5	0.6716 (3)	0.25355 (18)	0.4048 (2)	0.0144 (6)

H5	0.74328	0.25135	0.380255	0.017*
C6	0.6561 (3)	0.1977 (2)	0.4878 (2)	0.0181 (6)
H6	0.715344	0.151857	0.528342	0.022*
C7	0.5366 (3)	0.22197 (19)	0.5000 (2)	0.0179 (6)
H7	0.50179	0.195138	0.549818	0.021*
C8	0.4783 (3)	0.29337 (19)	0.4246 (2)	0.0147 (6)
H8	0.397726	0.322723	0.415683	0.018*
C9	0.7111 (3)	0.4770 (2)	0.5497 (2)	0.0194 (7)
H9	0.699657	0.524867	0.497316	0.023*
C10	0.6239 (3)	0.4545 (2)	0.6037 (2)	0.0177 (6)
H10	0.543627	0.484821	0.593797	0.021*
C11	0.6767 (3)	0.3790 (2)	0.6750 (2)	0.0213 (7)
H11	0.638054	0.349734	0.72102	0.026*
C12	0.7972 (3)	0.3551 (2)	0.6656 (2)	0.0244 (7)
H12	0.853732	0.306978	0.704488	0.029*
C13	0.8192 (3)	0.4150 (2)	0.5880 (2)	0.0238 (7)
H13	0.892609	0.414117	0.565643	0.029*
C14	0.2895 (3)	0.35130 (19)	0.2068 (2)	0.0140 (6)
C15	0.1742 (3)	0.3995 (2)	0.2065 (2)	0.0156 (6)
H15	0.162618	0.466391	0.20834	0.019*
C16	0.0809 (3)	0.3289 (2)	0.2032 (2)	0.0209 (7)
H16	−0.003988	0.340458	0.203319	0.025*
C17	0.1353 (3)	0.2384 (2)	0.1995 (2)	0.0209 (7)
H17	0.092877	0.17904	0.196154	0.025*
C18	0.2636 (3)	0.2511 (2)	0.2016 (2)	0.0176 (6)
H18	0.322191	0.201926	0.199956	0.021*
C19	0.1889 (3)	0.3340 (2)	−0.0603 (2)	0.0208 (7)
H19	0.27249	0.347447	−0.062494	0.025*
C20	0.1376 (3)	0.2426 (2)	−0.0567 (2)	0.0197 (7)
H20	0.180714	0.183946	−0.055911	0.024*
C21	0.0111 (3)	0.2533 (2)	−0.0543 (2)	0.0201 (7)
H21	−0.04585	0.203153	−0.051976	0.024*
C22	−0.0160 (3)	0.3527 (2)	−0.0560 (2)	0.0205 (7)
H22	−0.094107	0.380448	−0.054727	0.025*
C23	0.0941 (3)	0.4027 (2)	−0.0601 (2)	0.0201 (7)
H23	0.102955	0.469885	−0.062245	0.024*
Fe1	0.65338 (4)	0.33776 (3)	0.52362 (3)	0.01289 (11)
Fe2	0.13851 (4)	0.31869 (3)	0.07340 (3)	0.01267 (11)
S1	0.38303 (7)	0.48864 (5)	0.11639 (6)	0.01651 (16)
S2	0.57355 (7)	0.52584 (5)	0.15958 (6)	0.01945 (17)
S3	0.79134 (7)	0.43455 (6)	0.30660 (7)	0.0298 (2)

Atomic displacement parameters (Å^2)

	U^{11}	U^{22}	U^{33}	U^{12}	U^{13}	U^{23}
C1	0.0184 (14)	0.0122 (13)	0.0083 (13)	−0.0008 (11)	0.0049 (12)	−0.0026 (11)
C2	0.0153 (14)	0.0121 (13)	0.0112 (13)	−0.0026 (11)	0.0048 (12)	−0.0021 (11)
C3	0.0166 (14)	0.0213 (15)	0.0137 (14)	−0.0011 (12)	0.0044 (12)	−0.0015 (13)
C4	0.0112 (13)	0.0131 (13)	0.0116 (13)	−0.0025 (11)	0.0020 (11)	−0.0033 (12)
C5	0.0159 (14)	0.0118 (14)	0.0142 (14)	0.0005 (11)	0.0033 (12)	−0.0049 (12)
C6	0.0222 (16)	0.0124 (14)	0.0139 (14)	0.0024 (12)	−0.0019 (13)	−0.0014 (12)
C7	0.0227 (16)	0.0153 (14)	0.0143 (14)	−0.0074 (13)	0.0042 (13)	0.0004 (12)

C8	0.0121 (14)	0.0176 (15)	0.0123 (14)	−0.0036 (12)	0.0014 (12)	−0.0007 (12)
C9	0.0273 (16)	0.0136 (14)	0.0138 (14)	−0.0035 (13)	0.0018 (13)	−0.0043 (12)
C10	0.0209 (15)	0.0171 (15)	0.0135 (14)	0.0040 (12)	0.0033 (12)	−0.0076 (12)
C11	0.0293 (17)	0.0231 (16)	0.0090 (14)	0.0015 (14)	0.0029 (13)	−0.0036 (13)
C12	0.0226 (16)	0.0236 (17)	0.0182 (16)	0.0058 (14)	−0.0052 (13)	−0.0077 (14)
C13	0.0171 (16)	0.0279 (17)	0.0228 (16)	−0.0058 (13)	0.0016 (13)	−0.0127 (15)
C14	0.0145 (14)	0.0160 (14)	0.0083 (13)	0.0002 (12)	−0.0005 (11)	0.0012 (12)
C15	0.0150 (14)	0.0217 (15)	0.0082 (13)	−0.0019 (12)	0.0010 (12)	−0.0047 (12)
C16	0.0170 (15)	0.0377 (19)	0.0094 (13)	−0.0040 (14)	0.0061 (12)	−0.0037 (14)
C17	0.0213 (16)	0.0284 (17)	0.0104 (14)	−0.0082 (14)	0.0015 (13)	0.0049 (13)
C18	0.0206 (15)	0.0150 (15)	0.0137 (14)	−0.0014 (12)	0.0010 (12)	0.0012 (12)
C19	0.0207 (16)	0.0318 (18)	0.0088 (13)	−0.0042 (14)	0.0033 (12)	−0.0029 (13)
C20	0.0248 (16)	0.0210 (16)	0.0125 (14)	0.0025 (13)	0.0051 (13)	−0.0048 (13)
C21	0.0200 (15)	0.0247 (16)	0.0105 (14)	−0.0069 (13)	−0.0020 (12)	−0.0014 (13)
C22	0.0156 (15)	0.0280 (17)	0.0132 (14)	0.0013 (13)	−0.0016 (12)	−0.0006 (13)
C23	0.0244 (17)	0.0195 (15)	0.0112 (14)	−0.0047 (13)	−0.0011 (13)	0.0032 (13)
Fe1	0.0142 (2)	0.0121 (2)	0.0100 (2)	0.00071 (16)	0.00085 (17)	−0.00160 (16)
Fe2	0.0116 (2)	0.0164 (2)	0.00860 (19)	−0.00166 (16)	0.00152 (16)	0.00006 (17)
S1	0.0167 (4)	0.0158 (4)	0.0143 (3)	−0.0020 (3)	0.0014 (3)	0.0036 (3)
S2	0.0181 (4)	0.0207 (4)	0.0178 (4)	−0.0060 (3)	0.0035 (3)	0.0045 (3)
S3	0.0138 (4)	0.0402 (5)	0.0334 (5)	−0.0021 (4)	0.0051 (4)	0.0137 (4)

Geometric parameters (Å, º)

C1—C2	1.379 (4)	C12—H12	0.95
C1—C14	1.470 (4)	C13—Fe1	2.055 (3)
C1—S1	1.726 (3)	C13—H13	0.95
C2—C3	1.449 (4)	C14—C18	1.433 (4)
C2—C4	1.472 (4)	C14—C15	1.438 (4)
C3—S3	1.661 (3)	C14—Fe2	2.053 (3)
C3—S2	1.736 (3)	C15—C16	1.418 (4)
C4—C5	1.436 (4)	C15—Fe2	2.040 (3)
C4—C8	1.439 (4)	C15—H15	0.95
C4—Fe1	2.072 (3)	C16—C17	1.412 (4)
C5—C6	1.417 (4)	C16—Fe2	2.042 (3)
C5—Fe1	2.046 (3)	C16—H16	0.95
C5—H5	0.95	C17—C18	1.416 (4)
C6—C7	1.420 (4)	C17—Fe2	2.041 (3)
C6—Fe1	2.028 (3)	C17—H17	0.95
C6—H6	0.95	C18—Fe2	2.048 (3)
C7—C8	1.418 (4)	C18—H18	0.95
C7—Fe1	2.033 (3)	C19—C20	1.410 (4)
C7—H7	0.95	C19—C23	1.422 (4)
C8—Fe1	2.045 (3)	C19—Fe2	2.053 (3)
C8—H8	0.95	C19—H19	0.95
C9—C10	1.415 (4)	C20—C21	1.412 (4)
C9—C13	1.428 (4)	C20—Fe2	2.041 (3)
C9—Fe1	2.051 (3)	C20—H20	0.95
C9—H9	0.95	C21—C22	1.426 (4)
C10—C11	1.418 (4)	C21—Fe2	2.040 (3)
C10—Fe1	2.043 (3)	C21—H21	0.95
C10—H10	0.95	C22—C23	1.418 (4)

C11—C12	1.415 (4)	C22—Fe2	2.047 (3)
C11—Fe1	2.043 (3)	C22—H22	0.95
C11—H11	0.95	C23—Fe2	2.063 (3)
C12—C13	1.419 (5)	C23—H23	0.95
C12—Fe1	2.050 (3)	S1—S2	2.0525 (10)

C2—C1—C14	128.6 (2)	C20—C21—C22	107.8 (3)
C2—C1—S1	119.1 (2)	C20—C21—Fe2	69.79 (16)
C14—C1—S1	112.35 (19)	C22—C21—Fe2	69.84 (16)
C1—C2—C3	115.4 (2)	C20—C21—H21	126.1
C1—C2—C4	122.2 (2)	C22—C21—H21	126.1
C3—C2—C4	122.3 (2)	Fe2—C21—H21	125.9
C2—C3—S3	131.5 (2)	C23—C22—C21	108.0 (3)
C2—C3—S2	113.9 (2)	C23—C22—Fe2	70.40 (16)
S3—C3—S2	114.56 (17)	C21—C22—Fe2	69.32 (15)
C5—C4—C8	106.3 (2)	C23—C22—H22	126
C5—C4—C2	128.3 (3)	C21—C22—H22	126
C8—C4—C2	125.4 (2)	Fe2—C22—H22	125.8
C5—C4—Fe1	68.61 (15)	C22—C23—C19	107.5 (3)
C8—C4—Fe1	68.57 (15)	C22—C23—Fe2	69.22 (17)
C2—C4—Fe1	129.04 (19)	C19—C23—Fe2	69.44 (16)
C6—C5—C4	108.7 (3)	C22—C23—H23	126.2
C6—C5—Fe1	68.97 (16)	C19—C23—H23	126.2
C4—C5—Fe1	70.56 (15)	Fe2—C23—H23	126.7
C6—C5—H5	125.6	C6—Fe1—C7	40.94 (12)
C4—C5—H5	125.6	C6—Fe1—C11	120.22 (12)
Fe1—C5—H5	126.4	C7—Fe1—C11	104.35 (13)
C5—C6—C7	108.3 (2)	C6—Fe1—C10	156.95 (13)
C5—C6—Fe1	70.32 (16)	C7—Fe1—C10	121.46 (12)
C7—C6—Fe1	69.73 (16)	C11—Fe1—C10	40.62 (11)
C5—C6—H6	125.9	C6—Fe1—C8	68.57 (11)
C7—C6—H6	125.9	C7—Fe1—C8	40.69 (11)
Fe1—C6—H6	125.7	C11—Fe1—C8	120.94 (12)
C8—C7—C6	107.9 (3)	C10—Fe1—C8	107.83 (11)
C8—C7—Fe1	70.12 (15)	C6—Fe1—C5	40.71 (11)
C6—C7—Fe1	69.33 (16)	C7—Fe1—C5	68.62 (12)
C8—C7—H7	126.1	C11—Fe1—C5	157.56 (11)
C6—C7—H7	126.1	C10—Fe1—C5	161.08 (11)
Fe1—C7—H7	126.1	C8—Fe1—C5	68.41 (11)
C7—C8—C4	108.9 (2)	C6—Fe1—C12	105.52 (12)
C7—C8—Fe1	69.19 (15)	C7—Fe1—C12	119.71 (12)
C4—C8—Fe1	70.53 (15)	C11—Fe1—C12	40.44 (12)
C7—C8—H8	125.6	C10—Fe1—C12	68.02 (12)
C4—C8—H8	125.6	C8—Fe1—C12	156.00 (13)
Fe1—C8—H8	126.3	C5—Fe1—C12	123.14 (12)
C10—C9—C13	107.9 (3)	C6—Fe1—C9	159.73 (13)
C10—C9—Fe1	69.51 (16)	C7—Fe1—C9	159.11 (12)
C13—C9—Fe1	69.81 (16)	C11—Fe1—C9	68.25 (12)
C10—C9—H9	126.1	C10—Fe1—C9	40.44 (12)
C13—C9—H9	126.1	C8—Fe1—C9	125.06 (11)
Fe1—C9—H9	126.2	C5—Fe1—C9	125.45 (12)
C9—C10—C11	108.3 (3)	C12—Fe1—C9	68.12 (12)

C9—C10—Fe1	70.06 (16)	C6—Fe1—C13	122.13 (12)
C11—C10—Fe1	69.68 (16)	C7—Fe1—C13	156.59 (12)
C9—C10—H10	125.8	C11—Fe1—C13	68.25 (13)
C11—C10—H10	125.8	C10—Fe1—C13	68.21 (12)
Fe1—C10—H10	126	C8—Fe1—C13	162.09 (12)
C12—C11—C10	107.8 (3)	C5—Fe1—C13	109.31 (12)
C12—C11—Fe1	70.04 (18)	C12—Fe1—C13	40.45 (13)
C10—C11—Fe1	69.70 (16)	C9—Fe1—C13	40.70 (12)
C12—C11—H11	126.1	C6—Fe1—C4	68.90 (11)
C10—C11—H11	126.1	C7—Fe1—C4	68.94 (11)
Fe1—C11—H11	125.8	C11—Fe1—C4	158.45 (12)
C11—C12—C13	108.4 (3)	C10—Fe1—C4	124.24 (11)
C11—C12—Fe1	69.52 (16)	C8—Fe1—C4	40.90 (11)
C13—C12—Fe1	69.96 (17)	C5—Fe1—C4	40.83 (10)
C11—C12—H12	125.8	C12—Fe1—C4	160.70 (12)
C13—C12—H12	125.8	C9—Fe1—C4	110.39 (11)
Fe1—C12—H12	126.3	C13—Fe1—C4	125.76 (12)
C12—C13—C9	107.5 (3)	C21—Fe2—C15	149.91 (12)
C12—C13—Fe1	69.59 (17)	C21—Fe2—C20	40.48 (12)
C9—C13—Fe1	69.49 (16)	C15—Fe2—C20	169.40 (12)
C12—C13—H13	126.2	C21—Fe2—C17	104.53 (12)
C9—C13—H13	126.2	C15—Fe2—C17	68.54 (12)
Fe1—C13—H13	126.3	C20—Fe2—C17	114.90 (12)
C18—C14—C15	107.5 (2)	C21—Fe2—C16	115.13 (12)
C18—C14—C1	127.9 (3)	C15—Fe2—C16	40.64 (11)
C15—C14—C1	124.6 (2)	C20—Fe2—C16	147.95 (12)
C18—C14—Fe2	69.38 (15)	C17—Fe2—C16	40.47 (12)
C15—C14—Fe2	68.97 (15)	C21—Fe2—C22	40.84 (11)
C1—C14—Fe2	126.0 (2)	C15—Fe2—C22	118.59 (12)
C16—C15—C14	107.5 (3)	C20—Fe2—C22	68.24 (12)
C16—C15—Fe2	69.75 (16)	C17—Fe2—C22	126.44 (12)
C14—C15—Fe2	69.89 (16)	C16—Fe2—C22	107.48 (12)
C16—C15—H15	126.2	C21—Fe2—C18	125.60 (11)
C14—C15—H15	126.2	C15—Fe2—C18	68.99 (12)
Fe2—C15—H15	125.7	C20—Fe2—C18	106.65 (12)
C17—C16—C15	108.6 (3)	C17—Fe2—C18	40.51 (11)
C17—C16—Fe2	69.72 (17)	C16—Fe2—C18	68.30 (12)
C15—C16—Fe2	69.60 (16)	C22—Fe2—C18	163.99 (11)
C17—C16—H16	125.7	C21—Fe2—C14	165.36 (11)
C15—C16—H16	125.7	C15—Fe2—C14	41.13 (11)
Fe2—C16—H16	126.6	C20—Fe2—C14	129.44 (12)
C16—C17—C18	108.6 (3)	C17—Fe2—C14	68.42 (11)
C16—C17—Fe2	69.82 (17)	C16—Fe2—C14	68.46 (11)
C18—C17—Fe2	70.02 (17)	C22—Fe2—C14	153.54 (11)
C16—C17—H17	125.7	C18—Fe2—C14	40.92 (11)
C18—C17—H17	125.7	C21—Fe2—C19	67.94 (12)
Fe2—C17—H17	126	C15—Fe2—C19	132.43 (12)
C17—C18—C14	107.8 (3)	C20—Fe2—C19	40.29 (12)
C17—C18—Fe2	69.47 (16)	C17—Fe2—C19	149.68 (12)
C14—C18—Fe2	69.70 (15)	C16—Fe2—C19	169.72 (12)
C17—C18—H18	126.1	C22—Fe2—C19	67.94 (12)
C14—C18—H18	126.1	C18—Fe2—C19	118.68 (12)

Fe2—C18—H18	126.3	C14—Fe2—C19	111.23 (12)
C20—C19—C23	108.3 (3)	C21—Fe2—C23	68.23 (12)
C20—C19—Fe2	69.38 (17)	C15—Fe2—C23	111.25 (12)
C23—C19—Fe2	70.14 (17)	C20—Fe2—C23	68.04 (12)
C20—C19—H19	125.8	C17—Fe2—C23	165.92 (12)
C23—C19—H19	125.8	C16—Fe2—C23	130.29 (12)
Fe2—C19—H19	126.2	C22—Fe2—C23	40.38 (12)
C19—C20—C21	108.3 (3)	C18—Fe2—C23	153.47 (12)
C19—C20—Fe2	70.33 (17)	C14—Fe2—C23	121.35 (11)
C21—C20—Fe2	69.73 (17)	C19—Fe2—C23	40.42 (12)
C19—C20—H20	125.8	C1—S1—S2	93.98 (10)
C21—C20—H20	125.8	C3—S2—S1	97.18 (10)
Fe2—C20—H20	125.7		

C14—C1—C2—C3	176.4 (3)	Fe1—C9—C13—C12	−59.4 (2)
S1—C1—C2—C3	−4.0 (3)	C10—C9—C13—Fe1	59.30 (19)
C14—C1—C2—C4	−6.1 (4)	C2—C1—C14—C18	−51.1 (4)
S1—C1—C2—C4	173.5 (2)	S1—C1—C14—C18	129.3 (3)
C1—C2—C3—S3	−174.6 (2)	C2—C1—C14—C15	130.3 (3)
C4—C2—C3—S3	7.9 (4)	S1—C1—C14—C15	−49.3 (3)
C1—C2—C3—S2	7.0 (3)	C2—C1—C14—Fe2	−141.9 (2)
C4—C2—C3—S2	−170.5 (2)	S1—C1—C14—Fe2	38.5 (3)
C1—C2—C4—C5	146.3 (3)	C18—C14—C15—C16	1.0 (3)
C3—C2—C4—C5	−36.4 (4)	C1—C14—C15—C16	179.8 (2)
C1—C2—C4—C8	−31.3 (4)	Fe2—C14—C15—C16	59.86 (19)
C3—C2—C4—C8	146.0 (3)	C18—C14—C15—Fe2	−58.88 (19)
C1—C2—C4—Fe1	−121.3 (3)	C1—C14—C15—Fe2	120.0 (3)
C3—C2—C4—Fe1	56.0 (4)	C14—C15—C16—C17	−1.0 (3)
C8—C4—C5—C6	0.1 (3)	Fe2—C15—C16—C17	59.0 (2)
C2—C4—C5—C6	−177.9 (3)	C14—C15—C16—Fe2	−59.95 (19)
Fe1—C4—C5—C6	58.56 (19)	C15—C16—C17—C18	0.6 (3)
C8—C4—C5—Fe1	−58.48 (18)	Fe2—C16—C17—C18	59.5 (2)
C2—C4—C5—Fe1	123.5 (3)	C15—C16—C17—Fe2	−58.91 (19)
C4—C5—C6—C7	0.1 (3)	C16—C17—C18—C14	0.0 (3)
Fe1—C5—C6—C7	59.64 (19)	Fe2—C17—C18—C14	59.40 (19)
C4—C5—C6—Fe1	−59.53 (18)	C16—C17—C18—Fe2	−59.4 (2)
C5—C6—C7—C8	−0.3 (3)	C15—C14—C18—C17	−0.6 (3)
Fe1—C6—C7—C8	59.76 (19)	C1—C14—C18—C17	−179.4 (3)
C5—C6—C7—Fe1	−60.01 (19)	Fe2—C14—C18—C17	−59.3 (2)
C6—C7—C8—C4	0.3 (3)	C15—C14—C18—Fe2	58.62 (19)
Fe1—C7—C8—C4	59.56 (19)	C1—C14—C18—Fe2	−120.2 (3)
C6—C7—C8—Fe1	−59.26 (19)	C23—C19—C20—C21	0.1 (3)
C5—C4—C8—C7	−0.2 (3)	Fe2—C19—C20—C21	59.6 (2)
C2—C4—C8—C7	177.8 (2)	C23—C19—C20—Fe2	−59.53 (19)
Fe1—C4—C8—C7	−58.74 (19)	C19—C20—C21—C22	−0.3 (3)
C5—C4—C8—Fe1	58.50 (17)	Fe2—C20—C21—C22	59.7 (2)
C2—C4—C8—Fe1	−123.5 (3)	C19—C20—C21—Fe2	−59.99 (19)
C13—C9—C10—C11	−0.1 (3)	C20—C21—C22—C23	0.3 (3)
Fe1—C9—C10—C11	59.39 (19)	Fe2—C21—C22—C23	60.0 (2)
C13—C9—C10—Fe1	−59.49 (19)	C20—C21—C22—Fe2	−59.7 (2)
C9—C10—C11—C12	0.3 (3)	C21—C22—C23—C19	−0.3 (3)
Fe1—C10—C11—C12	59.9 (2)	Fe2—C22—C23—C19	59.07 (19)

C9—C10—C11—Fe1	−59.62 (19)	C21—C22—C23—Fe2	−59.4 (2)
C10—C11—C12—C13	−0.4 (3)	C20—C19—C23—C22	0.1 (3)
Fe1—C11—C12—C13	59.3 (2)	Fe2—C19—C23—C22	−58.9 (2)
C10—C11—C12—Fe1	−59.69 (19)	C20—C19—C23—Fe2	59.06 (19)
C11—C12—C13—C9	0.3 (3)	C2—C1—S1—S2	−0.3 (2)
Fe1—C12—C13—C9	59.36 (19)	C14—C1—S1—S2	179.37 (19)
C11—C12—C13—Fe1	−59.1 (2)	C2—C3—S2—S1	−6.2 (2)
C10—C9—C13—C12	−0.1 (3)	S3—C3—S2—S1	175.17 (15)
